# An Improved FCM Medical Image Segmentation Algorithm Based on MMTD

**DOI:** 10.1155/2014/690349

**Published:** 2014-02-04

**Authors:** Ningning Zhou, Tingting Yang, Shaobai Zhang

**Affiliations:** ^1^Computer School, Nanjing University of Posts and Telecommunications, Nanjing 210003, China; ^2^NARI Technology Development Co., Ltd., Nanjing 210061, China

## Abstract

Image segmentation plays an important role in medical image processing. Fuzzy c-means (FCM) is one of the popular clustering algorithms for medical image segmentation. But FCM is highly vulnerable to noise due to not considering the spatial information in image segmentation. This paper introduces medium mathematics system which is employed to process fuzzy information for image segmentation. It establishes the medium similarity measure based on the measure of medium truth degree (MMTD) and uses the correlation of the pixel and its neighbors to define the medium membership function. An improved FCM medical image segmentation algorithm based on MMTD which takes some spatial features into account is proposed in this paper. The experimental results show that the proposed algorithm is more antinoise than the standard FCM, with more certainty and less fuzziness. This will lead to its practicable and effective applications in medical image segmentation.

## 1. Introduction

Image segmentation is the procedure in which the original image is partitioned into homogeneous regions and plays an important role in medical image processing. As the imaging mechanism and the tissues of medical images are different, medical images are easily affected by noise, field migration effect, and tissue movement. Compared with the common images, medical images have more nonuniformity and fuzziness. Several popular clustering techniques for segmentation are available. Fuzzy c-means (FCM) is one such soft segmentation technique applicable for medical images. The performance of this method to obtain an optimal solution depends on the initial positions of the centers of the clusters, the measure of membership degree for each data point, and so on. In the standard FCM, the centers are initialized randomly and the measure of membership only uses the gray feature. This leads to be quite time-consuming and be sensitive to noise. For years, many research efforts have been made towards effective FCM image segmentation approaches. In order to accelerate the segmentation process, some approaches [1–4] focus on how to initialize the centers of required clusters. As noise is always emerged in the medical images, some FCM segmentation approaches [5, 6] which have less sensitivity to noise are also presented. Moreover, in order to overcome the defect that the FCM is easy to fall into local optimal solution, some scholars combine the FCM and other mathematical approaches [7, 8]. Nevertheless, due to their complexities and poor portabilities, effective medical image segmentation methods are yet to be seen.

This paper introduces medium mathematics system which is employed to process fuzzy information for image segmentation. It establishes the medium similarity measure based on the measure of medium truth degree (MMTD) and uses the correlation of the pixel and its neighbors to define the medium membership function. An improved FCM medical image segmentation algorithm based on MMTD which takes some spatial features into account is proposed in this paper. The experimental results show that the proposed algorithm is more antinoise than the standard FCM, with more certainty and less fuzziness.

## 2. The Medium Mathematics System

Medium principle was established by Zhu and Xiao Xi-an in the 1980s who devised medium logic tools [9] to build the medium mathematics system [10], the corner stone of which is medium axiomatic sets.

### 2.1. Basic Symbols in Medium Mathematics System

In medium mathematics system [10], predicate (conception or quality) is represented by *P* and any variable is denoted as *x*, with *x* completely possessing quality *P* being described as *P*(*x*). The “*╕*” symbol stands for inverse opposite negative and it is termed as “opposite to.” The inverse opposite of predicate is denoted as *╕P* and then the concept of a pair of inverse opposite is represented by both *P* and *╕P*. Symbol “~” denotes fuzzy negative which reflects the medium state of “either or” or “both this and that” in opposite transition process. The fuzzy negative profoundly reflects fuzziness; “≺” is a truth-value degree connective which describes the difference between two propositions.

### 2.2. Measure of Medium Truth Degree

**Figure 1:**
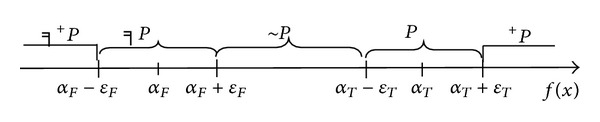
Relation between numerical value areas and predication.

According to the concept of super state [11, 12], the numerical value area of generally applicable quantification is divided into five areas corresponding to the predicate truth scale, namely, *╕* ^+^ *P*, *╕P*, ~*P*, *P*, and ^+^ *P*, as shown in Figure 1. In “True” numerical value area T, *α*_*T*_ is *ε*_*T*_ standard scale of predicate *P*; in “False” numerical value area F, *α*_*F*_ is *ε*_*F*_ standard scale of predicate *╕P*.

Individual truth scale in each numerical value area can be calculated by the distance ratio *h*_*T*_(*f*(*x*)) (or *h*_*F*_(*f*(*x*))) [11] which relates to *P* (or *╕P*).

## 3. The MMTDFCM Medical Image Segmentation Algorithm

### 3.1. Medium Similarity Measure of Two Pixels

Similarity degree between two pixels can be scaled by the difference or the ratio of the gray level between pixels. This paper firstly discusses a new similarity measure based on MMTD [11, 12].

**Figure 2:**
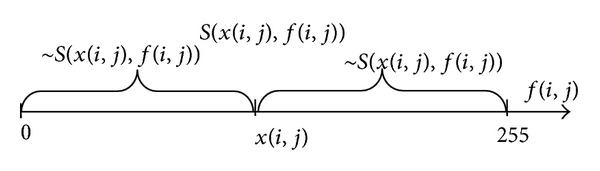
Relation between the gray level of pixels *x*(*i*, *j*)  and  *f*(*i*, *j*) and predicate similar.

Given there are two pixels *x*(*i*, *j*) and*f*(*i*, *j*) in grey images whose range of gray level is 0 ~ 255. The two pixels may be in the same image but have different locations or they belong to different images but have the same coordinate. First, we relate the gray level of pixels to a number axis. Second, predicate *S*(*x*(*i*, *j*), *f*(*i*, *j*)) represents that *x*(*i*, *j*) is similar to *f*(*i*, *j*), *╕S*(*x*(*i*, *j*), *f*(*i*, *j*)) represents that *x*(*i*, *j*) is different from*f*(*i*, *j*) and ~*S*(*x*(*i*, *j*), *f*(*i*, *j*)) transition, as shown in Figure 2. According to the measure of medium truth degree, we give the definition of the similarity degree between *x*(*i*, *j*) and *f*(*i*, *j*).

**Definition 1**

For pixels *x*(*i*, *j*) and *f*(*i*, *j*), we define

(1) hh(f(i,j),x(i,j)) =12[h(f(i,j),x(i,j))+h(x(i,j),f(i,j))],

where

(2) $$\begin{array}{c} h\left(f\left(i,j\right),x\left(i,j\right)\right)=\{\begin{array}{cc} \frac{d\left(f\left(i,j\right),-1\right)}{d\left(x\left(i,j\right),-1\right)} & f\left(i,j\right)<x\left(i,j\right)\\ 1 & f\left(i,j\right)=x\left(i,j\right)\\ \frac{d\left(f\left(i,j\right),256\right)}{d\left(x\left(i,j\right),256\right)}\; & f\left(i,j\right)>x\left(i,j\right), \end{array} \end{array}$$

as the medium similarity measure of two pixels, where *d*(*a*, *b*) is the Euclidean distance between *a* and *b*. Under the one-dimensional circumstance, *d*(*a*, *b*) = |*a* − *b*|. The larger the value is, the higher the similarity degree between *f*(*i*, *j*) and *x*(*i*, *j*) becomes. When it equals 1, it shows that the pixels *f*(*i*, *j*) and *x*(*i*, *j*) are similar. The smaller the value is, the lower the similarity degree between *f*(*i*, *j*) and *x*(*i*, *j*) becomes. When it equals 0, it shows that the pixels *f*(*i*, *j*) and *x*(*i*, *j*) are different.

### 3.2. Medium Membership Function

There are abundant information reflected by gray, edge, texture, and space in an image. Images are always polluted during image acquisition and transmission, so the noise should be removed before the further image segmentation. Standard FCM image segmentation approach which only takes the gray feature into account and ignores the other features is very sensitive to noise. This leads to some wrong classifications. For example, some pixels which should belong to the homogeneous region are separated. In order to improve the antinoise and the effect of the segmentation, we introduce the spatial feature and the correlation between the pixel and its neighbors to image segmentation.

**Figure 3:**
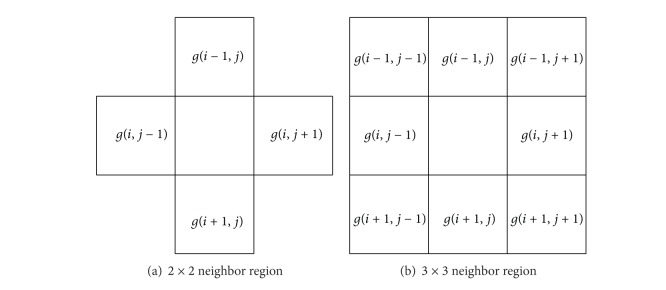
Neighbor regions of *g*(*i*, *j*).

Let *G*_*M*×*N*_ = [*g*(*i*, *j*)]_*M*×*N*_ and denote an image of *M* × *N* pixels. The image has *L* gray levels; that is, *G* = {0, 1, 2, …, *L* − 1}. The grey level of pixel at coordinate (*i*, *j*) is expressed as *g*(*i*, *j*). Take a 2 × 2 slip window whose center pixel is *g*(*i*, *j*). Define the 2 × 2 neighbor region which includes the four regions *g*(*i* − 1, *j*), *g*(*i*, *j* − 1), *g*(*i*, *j* + 1), and  *g*(*i* + 1, *j*) as shown in Figure 3(a) and the 3 × 3 neighbor region which includes the 8 regions *g*(*i* − 1, *j* − 1), *g*(*i* − 1, *j*), *g*(*i* − 1, *j* + 1), *g*(*i*, *j* − 1), *g*(*i*, *j* + 1), *g*(*i* + 1, *j* − 1), *g*(*i* + 1, *j*), and *g*(*i* + 1, *j* + 1) as shown in Figure 3(b).

In standard FCM image segmentation approach, *u*_*ik*_ only related to gray feature is the fuzzy membership between the clustering sample pixel *x*_*k*_ and the clustering centre *v*_*i*_. In fact, no matter wherever the sample pixel is in the even region or region polluted by noise or the edge region, the influence of other pixels on the sample pixel should be considered. Because of the strong relationship between the sample pixel and its neighbors, we use the relationship to estimate the fuzzy membership in order to reduce the influence of noise and other uncorrelated pixels.

In grey image *G*_*M*×*N*_ = [*g*(*i*, *j*)]_*M*×*N*_, take a 3 × 3 slip window and regard the center pixel *g*(*i*, *j*) as the sample pixel. According to (1), we can get the medium similarity measure of the sample pixel *g*(*i*, *j*) and its neighbors. The larger the value is, the higher the similarity degree between the sample pixel *g*(*i*, *j*) and its neighbors becomes. The smaller the value is, the lower the similarity degree between the sample pixel *g*(*i*, *j*) and its neighbors becomes. Let $\bar{h}(i,j)$ denote the mean similarity degree between the sample pixel *g*(*i*, *j*) and its 8 neighbors, as follows:

(3) h¯(i,j)={h[g(i,j),g(i−1,j−1)] +h[g(i,j),g(i−1,j)] +h[g(i,j),g(i−1,j+1)] +h[g(i,j),g(i+1,j−1)] +h[g(i,j),g(i+1,j)] +h[g(i,j),g(i+1,j+1)] +h[g(i,j),g(i,j−1)] +h[g(i,j),g(i,j+1)]}/8.

$\bar{h}(i,j)$ reflects the similarity degree between the sample pixel *g*(*i*, *j*) and its neighbors. When the sample pixel *g*(*i*, *j*) is a normal pixel, the relationship between the sample pixel *g*(*i*, *j*) and its neighbors is strong and the value of $\bar{h}(i,j)$ is high. When the sample pixel *g*(*i*, *j*) is a noise pixel, the relationship between the sample pixel *g*(*i*, *j*) and its neighbors is weak and the value of $\bar{h}(i,j)$ is low. The strong related pixels play the key role in the classification while the weak related pixels have relatively small impact on the classification. As a result, we use $\bar{h}(i,j)$ to modify membership function to reduce the influence of noise and uncorrelated pixels and improve the accuracy of classification.

Firstly, according to (3), compute and row the $\bar{h}(i,j)$ of every sample pixel *g*(*i*, *j*), and then we get one-dimensional matrix ${\left[\bar{h}(k)\right]}_{M\times N}$, where $\bar{h}(k)$ represents the mean similarity degree between the *k*th pixel *x*_*k*_ and its 8 neighbors. Secondly row the gray image *G*_*M*×*N*_ = [*g*(*i*, *j*)]_*M*×*N*_, and then use the classical FCM to calculate the fuzzy membership *u*_*ik*_ and the mean fuzzy membership ${\bar{u}}_{ik}$ between the *k*th pixel *x*_*k*_ and its neighbors:

(4) $$\begin{array}{c} {\bar{u}}_{ik}=\sum_{j\in NB(k)}u_{ij}, \end{array}$$

where *NB*(*k*) represents the 3 × 3 neighbor region of center pixel *x*_*k*_.

**Definition 2**

For pixel  *x*_*k*_, we define

(5) $$\begin{array}{c} u_{ik}^{\prime }=\overset{-}{h}\left(k\right){\bar{u}}_{ik}+\left[1-\overset{-}{h}\left(k\right)\right]u_{ik} \end{array}$$

as the medium membership function.

The *u*_*ik*_′ which considers the spatial information, the similarity degree, and the correlation between the sample pixel and its neighbors can help classify the sample pixel into the correct region.

When the sample pixel *x*_*k*_ has a high correlation with pixels in its 3 × 3 neighbor region, medium membership grade *u*_*ik*_′ is mainly judged by the mean fuzzy membership ${\bar{u}}_{ik}$. It shows that the sample pixel *x*_*k*_ has a high medium membership to class *i*. Otherwise, when the sample pixel *x*_*k*_ has a low correlation with pixels in its 3 × 3 neighbor region, medium membership grade *u*_*ik*_′ is mainly judged by the fuzzy membership grade *u*_*ik*_. This is more reasonable. The classification approach which uses the medium membership instead of fuzzy membership to get the cluster centers by iterative computation is more likely to get the correct classification.

### 3.3. Main Steps of the MMTDFCM Medical Image Segmentation Algorithm

The computation of the MMTDFCM medical image segmentation algorithm is implemented in the following steps.

(1) Initialization: set the fuzzy weighting exponent *m* > 1 and iterative threshold *ε* > 0. Set the cluster number *c*  (2 ≤ *c* ≤ *n*). Initialize the cluster center *V* ^0^ and set iterative counter *l* = 0.
(2) According to (3), compute and row the $\bar{h}(i,j)$ of every sample pixel *g*(*i*, *j*), and we can get one-dimensional matrix ${\left[\bar{h}(k)\right]}_{M\times N}$, where $\bar{h}(k)$ represents the mean similarity degree between the *k*th pixel *x*_*k*_ and its 8 neighbors and then row the gray image *G*_*M*×*N*_ = [*g*(*i*, *j*)]_*M*×*N*_, and let *n* = *M* × *N*.According to (6), compute the fuzzy membership *u*_*ik*_. To for all *i*, *k*, if ∃*d*_*ik*_^(*l*)^ > 0,
  (6) $$\begin{array}{c} u_{ik}^{\left(l\right)}={\left\{{\sum_{j=1}^{c}\left[\frac{d_{ik}^{\left(l\right)}}{d_{jk}^{\left(l\right)}}\right]}^{2/\left(m-1\right)}\right\}}^{-1}. \end{array}$$
  If ∃*i*, *r*, *d*_*ik*_^(*l*)^ = 0, then *u*_*ir*_^(*l*)^ = 1, and if *j* ≠ *i*, then *u*_*jr*_^(*l*)^ = 0.
(3) According to (4), compute the mean fuzzy membership ${\bar{u}}_{ik}$between the pixel and its neighbors.
(4) According to (5), compute the medium membership *u*_*ik*_′ of every pixel.
(5) According to (7), update the cluster centre matrix *V* ^(*l*+1)^:
  (7) $$\begin{array}{c} v_{i}^{\left(l+1\right)}=\frac{\sum_{k=1}^{n}{\left({u^{\prime }}_{ik}^{\left(l\right)}\right)}^{m}\cdot x_{k}}{\sum_{k=1}^{n}{\left({u^{\prime }}_{ik}^{\left(l\right)}\right)}^{m}},\;i=1,2,\ldots ,c, \end{array}$$
  where *u*_*ik*_′ is the medium membership of sample pixel.
(6) If ||*V* ^(*l*+1)^ − *V* ^(*l*)^|| < *ε*, then go to the next step; else set *l* = *l* + 1, and go to Step (4); where ||·|| is a norm of a matrix.
(7) Defuzzification. Use the maximum membership function (as (8)) to reduce the fuzziness of every pixel:
  (8) $$\begin{array}{c} C_{k}=\text{ar}{\text{g}}_{i}\left\{\max\left(u_{ik}\right)\right\}\;\;\;\forall i,\forall k, \end{array}$$
  where  *C*_*k*_ represents the classification which the *k*th pixel belongs to.

### 3.4. Experimental Results

Peak-value signal-to-noise (PSNR), partition coefficient *vpc*, and partition entropy *vpe* are chosen as the criterions to evaluate the performance of the MMTDFCM method:

(9) $$\begin{array}{c} \text{PSNR}=10\;\text{lg}\frac{x_{\max}^{2}}{\left(1/\left(M\times N\right)\right)\sum_{i=1}^{M}\sum_{j=1}^{N}{\left[x\left(i,j\right)-y\left(i,j\right)\right]}^{2}}, \end{array}$$

where *x*(*i*, *j*) is the grey level of pixel in original image and *y*(*i*, *j*) is the gray level of pixel in processed image; *X*_max⁡_ is the maximum gray level of the image; *M* and *N* are the sizes of the image.

For a given cluster number *c* and membership matrix [*u*_*ik*_]_*c*×*n*_, partition coefficient *vpc* and partition entropy *vpe* are defined as follows:

(10) $$\begin{array}{c} vpc=\frac{1}{n}\sum_{i=1}^{c}\;\sum_{k=1}^{n}u_{ik}^{2}, \end{array}$$

(11) $$\begin{array}{c} vpe=-\frac{1}{n}\sum_{i=1}^{c}\;\sum_{k=1}^{n}(u_{ik}\log u_{ik}). \end{array}$$

When it gets the best cluster result, partition coefficient *vpc* will be the maximum and partition entropy *vpe* will be the minimum.

**Figure 4:**
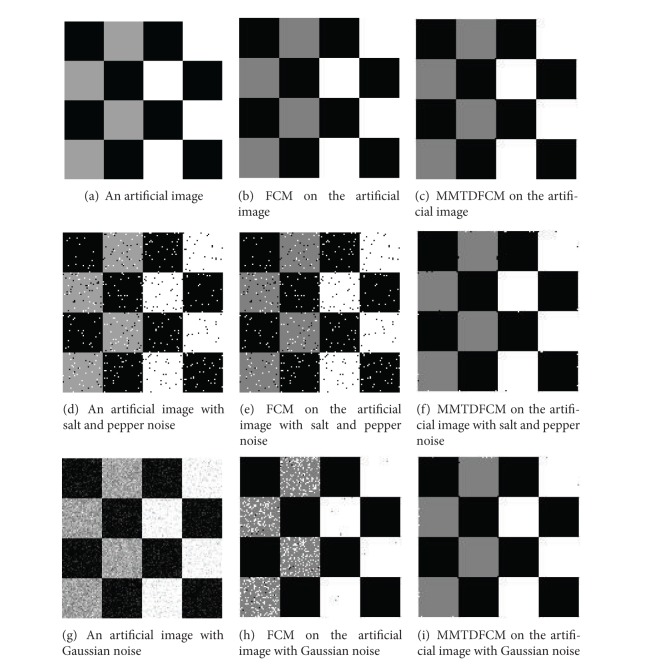
Segmentation of an artificial image (*c* = 3).

**Figure 5:**
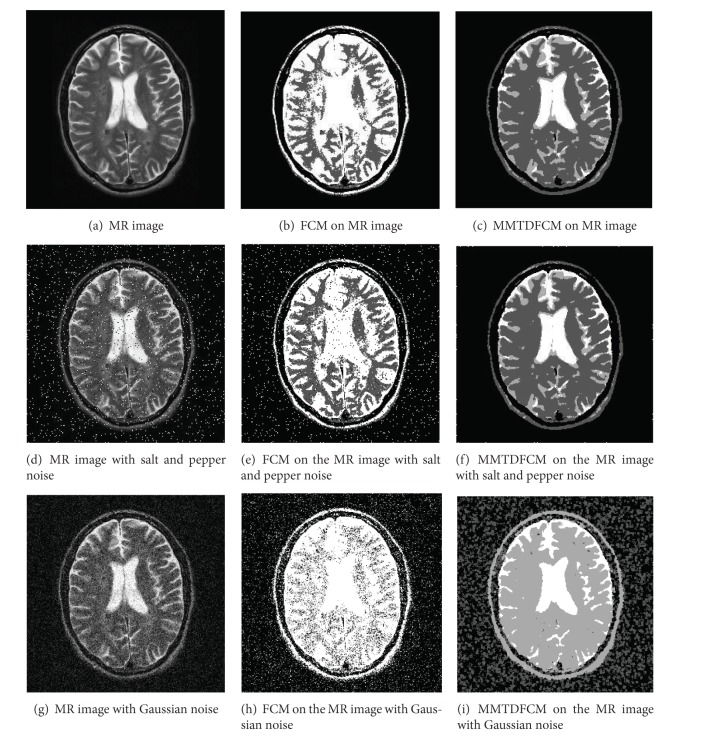
Segmentation of an MR image (*c* = 4).

An artificial image, an MR image, and an ROI image are chosen as experimental samples to verify the MMTDFCM algorithm described in this paper. Standard FCM is used in comparison with the experiment in order to evaluate the performance of the new algorithm in medical image segmentation. As the most common noises that emerged in medical images are Gaussian noise and salt and pepper noise, images with salt and pepper noise and Gaussian noise are chosen to test the antinoise ability of MMTDFCM. Fuzzy weighting exponent *m* controls the share degree of clusters, and, in practice, the best value range of *m* is considered as [1.5,2.5]. This also accords with conclusion of Pal's experiments [13]. Set the fuzzy weighting exponent *m* = 2 and iterative threshold *ε* = 0.00001. The experimental results are shown in Figure 4, Figure 5, and Tables 1, 2, and 3.

The artificial image (128 × 128)  includes three gray levels: 0, 160, and 255, and we set the cluster number *c* = 3. The MR image (256 × 256) and the ROI image (128 × 128) include the background, white matter, gray matter, and cerebrospinal fluid, so we set the cluster number *c* = 4.

**Figure 6:**
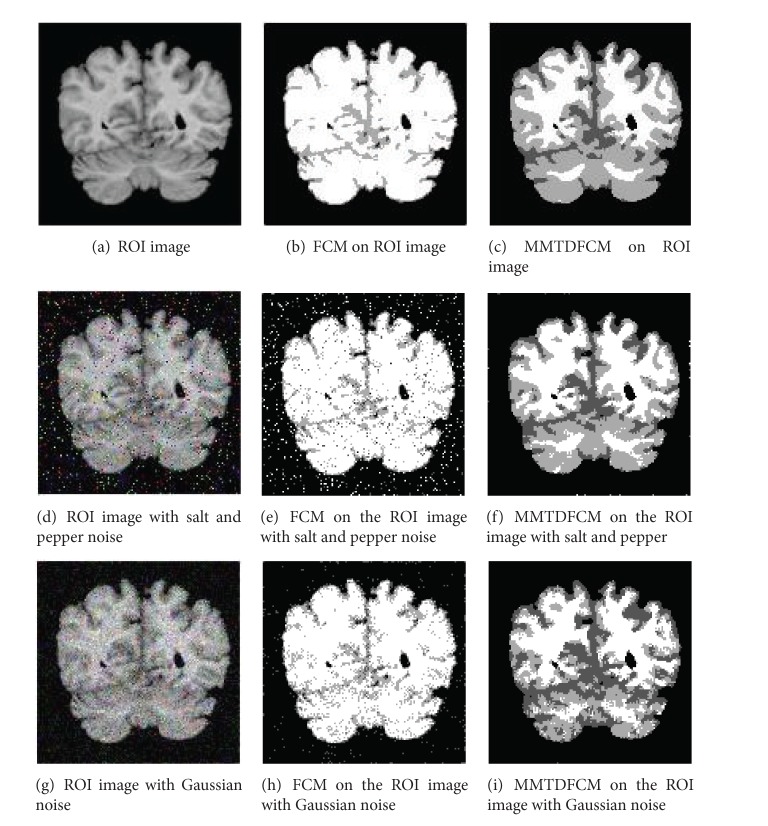
Segmentation of an ROI image (*c* = 4).

The performance of the new algorithm can be evaluated through both subjective visual and objective quality.

(1)* Subjective Visual Effect.* The experimental results as shown in Figures 4(b) and 4(c), Figures 5(b) and 5(c), and Figures 6(b) and 6(c) reveal that both FCM and MMTDFCM have the satisfied segmentation performance of an ideal images. Figures 4(e) and 4(f), Figures 5(e) and 5(f), and Figures 6(e) and 6(f) are the segmentation results of the images polluted by salt and pepper noise (*б* = 0.05). Figures 4(h) and 4(i), Figures 5(h) and 5(i), and Figures 6(h) and 6(i) are the segmentation results of the images polluted by Gaussian noise (*б* = 0.01). As it can be seen, due to the influence of the noise, standard FCM incorrectly classifies some pixels and the segmentation images by the standard FCM include much noise. The MMTDFCM which considers the spatial feature and the correlation between the pixel and its neighbors can get the higher quality of segmentation to the polluted images. MMTDFCM correctly classifies the background, white matter, gray matter, and cerebrospinal fluid. Moreover, the segmentation images have little noise. The segmentation effect is obviously better than standard FCM. Moreover, standard FCM is more dependent on the initial positions of the centers of the clusters.

(2)* Better Objective Quality*.  Table 1 to Table 3 show the segmentation accuracy of images with noise. From the tables, it can be seen that the PSNR of new algorithm is higher than the standard FCM. It shows that the MMTDFCM has higher antinoise performance. The results also show that, in many cases, the MMTDFCM has a larger partition coefficient *vpc* and a smaller partition entropy *vpe* than the standard FCM. It represents that the segmentation result by MMTDFCM has more certainty and less fuzziness.

## 4. Conclusion 

The standard FCM image segmentation algorithm is sensitive to noise because of not taking into account the spatial information of the image. This paper proposes an improved FCM medical segmentation algorithm based on MMTD. It uses medium similarity measure and correlation of the pixel and its neighbors and defines the medium membership function. Both the gray information and the spatial information are applied in MMTDFCM. As a result, the MMTDFCM can classify the pixels more correctly. The experimental results show that the proposed algorithm is more antinoise than the standard FCM image segmentation algorithm, with more certainty and less fuzziness. This will lead to its practicable and effective applications in medical image segmentation.

## Figures and Tables

**Table 1:** Segmentation accuracy of the artificial image.

Image	Algorithm	PSNR	*vpc *	*vpe *
Artificial image	FCM	23.7811	0.7329	0.4118
	MMTDFCM	23.7101	0.9582	0.0628

Artificial image with salt and pepper noise	FCM	15.7015	0.7526	0.3825
	MMTDFCM	22.4539	0.8967	0.1763

Artificial image with Gaussian noise	FCM	19.8219	0.7269	0.4392
	MMTDFCM	23.521	0.8655	0.2596

**Table 2:** Segmentation accuracy of the MR image.

Image	Algorithm	PSNR	*vpc *	*vpe *
MR image	FCM	11.4656	0.7902	0.4878
	MMTDFCM	20.9565	0.8424	0.2938

MR image with salt and pepper noise	FCM	11.2788	0.7291	0.5180
	MMTDFCM	20.0788	0.7841	0.4151

MR image with Gaussian noise	FCM	8.8638	0.5839	0.7885
	MMTDFCM	11.5931	0.4733	0.8957

**Table 3:** Segmentation accuracy of the ROI image.

Image	Algorithm	PSNR	*vpc *	*vpe *
ROI image	FCM	11.4767	0.8421	0.3182
	MMTDFCM	16.6042	0.7784	0.3948

ROI image with salt and pepper noise	FCM	10.7082	0.7678	0.4648
	MMTDFCM	15.5713	0.6677	0.6067

ROI image with Gaussian noise	FCM	11.8689	0.6147	0.6880
	MMTDFCM	15.8405	0.6786	0.5782
